# Academic performance and musculoskeletal pain in adolescents with uncorrected vision problems

**DOI:** 10.1186/s12887-024-04681-7

**Published:** 2024-03-21

**Authors:** Hanne-Mari Schiøtz Thorud, Prabeen Raj Mudvari, Helle K. Falkenberg

**Affiliations:** National Centre for Optics, Vision and Eye Care, Department of Optometry, Radiography and Lighting Design, University of South-Eastern Norway, Kongsberg, Norway

**Keywords:** Neck, Shoulder, Eye, Headache, Vision, Glasses, Children, Screen, Learning, Education

## Abstract

**Background:**

Undetected vision problems are common in school children, and a prevalence of up to 40% has previously been reported. Uncorrected vision and lack of optimal eye wear can have a significant impact on almost all aspects of everyday life, such as development and learning, academic performance, pain and discomfort, and quality of life. This study aimed to analyze the relationship between uncorrected vision problems, educational outcomes, and musculoskeletal pain symptoms.

**Methods:**

A total of 152 school children (15.1 ± 0.8 years, mean ± SD; 40% males) were included in the study. All participants were recruited from a free-of-charge school vision testing program in Kathmandu, Nepal. Academic grades were collected from the school records of the participants’ nationwide final grade examinations. A questionnaire was used to record the use of digital devices, screen time, and associated symptoms, including musculoskeletal pain (Wong-Baker FACES Pain Rating Scales).

**Results:**

A total of 61 children (40%) had uncorrected vision, with a cycloplegic refraction of SER − 0.53 ± 0.52 (mean ± SD). Children with uncorrected vision had significantly more third division grades (26 vs. 9%, *p* = 0.004) and shoulder pain in general/during screen use (66 vs. 43/40%, *p* = 0.008/0.003; 2.1/1.9 vs. 1.1/1.0 mean pain score, *p* = 0.002/0.001) compared with children with normal vision. Sex based subanalyses showed that only girls with uncorrected vision had more third division grades (25 vs. 4%, *p* = 0.006), and only boys with uncorrected vision had more shoulder pain in general/during screen use (76 vs. 28/31%, *p* < 0.001; 2.2/2.4 vs. 0.7 mean pain score, *p* < 0.001), compared with children with normal vision.

**Conclusions:**

The results of this study showed that even small refractive errors may impact educational outcomes and musculoskeletal pain in adolescents. Most of the participating children had low myopia, easily corrected with glasses. This suggests that regular eye examinations are important in school children, and there is a need for raised awareness among parents, and school- and healthcare personnel.

## Introduction

Vision problems, such as uncorrected refractive errors and lack of proper eye wear, are associated with huge economic costs and reduced quality of life globally [1, 2]. Undetected vision problems are common in school children, and a prevalence of 10–40% is reported in urban and suburban children in Asia, the USA, Australia, and North European countries [3–8]. In children, good vision is essential for all everyday activities and normal motor development [9]. Uncorrected vision problems affect fine and gross motor skills, including visual information processing skills, eye-hand coordination, and balance [9, 10]. Further, uncorrected vision problems may reduce the ability to concentrate, as well as sustain near tasks over time, such as reading and writing [4, 11–13]. Together, this may affect academic performance [12, 13]. Children with hyperopia (farsightedness) are less able to focus at near, and have a reduced ability to perform near work, including screen viewing, reading, and writing [13–15]. Children with myopia (nearsightedness) have reduced distance vision, affecting their ability to see the blackboard/smartboard in a classroom and navigate outside and in social settings [12, 14, 16–18]. Children with astigmatism have problems with blurred vision both at near and distance [14, 19, 20]. Providing eyeglasses and adherence to eye wear have been shown to improve educational outcomes in school children [16, 18, 21–24].

In addition, common vision problems are linked to symptoms of eyestrain, neck, shoulder, and back pain, and headache [3, 4, 11, 25–29]. Effortless visual performance requires optimal coordination between the eyes and the head-stabilizing musculature in the neck, shoulders and back. Uncorrected vision problems will increase the demand on the visual and musculoskeletal apparatus, thereby increasing the risk for pain symptoms in the eyes and neck, shoulder, and back. Increased neck- and shoulder pain may also provoke headache symptoms [26, 28, 30–36]. Headache, neck and back pain are leading causes of sickness absence globally, and the prevalence is increasing in the age group 10–24 years, highlighting the importance of preventive treatment also in children [37–40].

This study aimed to analyze the relationship between uncorrected vision problems, educational outcomes, and musculoskeletal pain symptoms.

## Methods

### Participants

**Fig. 1:**
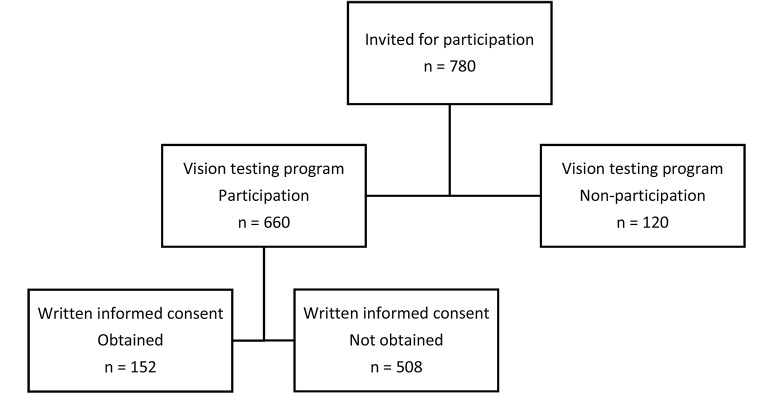
Flowchart of participant recruitment

This was a cross-sectional study in 14–16-year-old children at six secondary schools (two community schools, four private schools) in Kathmandu district/municipality, Nepal, in 2019. All 780 students aged 14–16 years at the six schools were invited to participate in the study. A free-of-charge school vision testing program was run by two experienced authorized optometrists (PRM and colleague) and three volunteers. A total of 660 students participated in the vision testing program, and written informed consent was obtained from 152 children and both their parents (Fig. 1). Children who needed an extended eye examination, or children who wanted their parents present, were examined by the optometrists (PRM and colleague) during after-school hours at an eye clinic in the vicinity of the schools. Children identified with ocular pathology, were referred to an ophthalmologist at the eye clinic.

### Eye examination

The eye examination was performed according to national and international clinical guidelines [41–43], in a separate room with adjustable lightning level at each school. A structured, age-appropriate patient history-taking [41–43] included vision problems during near and distance work (with and without current glasses), ocular and systemic health, and regular medication. The vision-related symptoms included headache, double vision, blurred vision at near/distance, and eye pain. The eye examination included: Habitual and best-corrected logMAR visual acuity (6 m and 40 cm), dominant eye (near/distance), refractive error with and without cycloplegia (Cyclopentolate Minims 1%); habitual cover test (near/distance), near point of convergence (NPC) (recorded as break (average of three)), binocular accommodation amplitude (AA BIN) (RAF-rule) [44]; motility, pupillary reflexes, ocular health (slit lamp examination, dilated direct ophthalmoscopy), anterior chamber depth (Van Herick technique). For analysis, cycloplegic spherical equivalent error (SER) was calculated in dioptres (D). Refractive errors were defined as emmetropia (− 0.50 D < SER < + 0.50 D), low myopia (-3.00 D < SER ≤ -0.50 D), moderate myopia (-5.00 D < SER ≤ − 3.00 D), high myopia (SER ≤ -5.00 D), low hyperopia (+ 0.50 D ≤ SER ≤ + 2.00 D), moderate hyperopia (+ 2.00 D < SER ≤ + 5.00 D), high hyperopia (SER > + 5.00 D), and anisometropia (difference between the eyes ≥ 1.00 D) [45, 46]. Normal visual acuity was defined as best corrected VA ≤ 0.0 logMAR. Distance heterophoria was defined as esophoria > 1 ΔBO (prism diopter base out) or exophoria > 2 ΔBI (prism diopter base in). Near heterophoria was defined as esophoria > 0 ΔBO or exophoria > 6 ΔBI. Normal NPC was defined as ≤ 10 cm and AA as > 8 D. Normal values are in line with previous studies [3, 47–49]. In this study, glasses for uncorrected refractive errors were recommended when the child had myopia and blurred distance vision, low hyperopia and headache, and moderate to high hyperopia. In addition, children already wearing glasses were recommended new glasses if there was a change of ≥ 0.50 SER [41, 42].

A self-reported questionnaire was used to elicit information about digital device usage and associated symptoms. Reported screen time included total smartphone, tablet, and computer use on a weekday and weekend (Saturday). Pain/discomfort in the eyes/head/neck/shoulders/lower back in general and during screen use, were scored using the Wong-Baker FACES Pain Rating Scale [50]. This scale uses faces to help children communicate about their pain. Each of the six faces (0, 2, 4, 6, 8, or 10 points) represents a different facial expression of pain. Continuous reading/screen viewing ability was recorded as ‘able to read or look at a screen for more than 30 min without having to take a break of more than 5 min because of tired eyes’. The project leader (PRM) instructed the children to complete their questionnaire individually. They were encouraged to answer honestly, were told that there were no correct or incorrect answers, and that they could take breaks or ask questions at any time. The questionnaire was in English. Nepalese 14–16-year-olds are mostly fluent in English, as English is mandatory as a second language from 1st grade, and many subjects are taught in English. A Nepalese translation was available from PRM if necessary. The participants took less than 30 min to complete the questionnaire.

### Academic performance

The academic grades were collected from the school records of the participants’ nationwide final grade examinations during spring 2019 (March/April), before the school vision testing program. The final examinations were English language, mother tongue (Nepali language), science, mathematics, social studies, and optional subjects. Academic performance was quantified using the average of all subject scores (score range: 0 to 100). The final grade rankings were distinction (≥ 80%), first division (≥ 60% to < 80%), second division (≥ 50% to < 60%), third division (≥ 45% to < 50%), and pass division (≥ 40% to < 45%).

### Statistical analysis

Sample size regarding musculoskeletal pain was calculated with a test power of 85%, a significance level of 5% (two-tailed), and standard errors based on previous pain recordings in adolescents [28]. The power analysis showed that 132 participants were sufficient to identify a mean difference of 1 on the Wong-Baker FACES Pain Rating Scale (0–10 points) [50] between two independent groups. Overall academic performance was the other main outcome variable, however, suitable scientific data/standard errors were unavailable to our knowledge. Sample size was therefore calculated with effect size (d = 0.5; medium), a test power of 85%, and a significance level of 5% (two-tailed). The power analysis showed that a sample of 146 participants was necessary to identify a significant difference in academic mean scores between two independent groups. Raw data were assessed for normality using Q-Q plots and the Shapiro-Wilk test. Differences between the prescription and control groups for continuous variables were tested by independent-samples t-tests. Paired sample t-test was used to compare dependent continuous variables. Chi-square independence tests were used to evaluate associations between categorical variables. Pearson’s correlation coefficient (*r*) was used to investigate associations between continuous variables. Point-biserial correlations were run to evaluate the relationship between categorical and continuous variables. To protect from Type I errors, Bonferroni corrections were performed for multiple comparisons when suitable [51]. Analysis and distributions using refractive errors included mainly right eye, as there were no significant differences between the right and left eyes (cycloplegic SER, paired-samples t-test; t(151) = 0.403, *p* = 0.687). The refractive error (cycloplegic) of the right eye and left eye was significantly and highly correlated (r(152) = 0.974, *p* < 0.001). A significant difference was set at *p* < 0.05 (two-tailed). Statistical analyses were performed in IBM SPSS Statistics (Version 28, US) and G*Power (Heinrich-Heine-Universität Düsseldorf, Germany).

## Results

The average age of the 152 included children was 15.1 ± 0.8 (SD) years, with 61 males (40%). Sixteen children (11%) reported allergies (dust, pollen). None of the children reported systemic diseases or use of regular medications. After the eye examination, the sample was divided into two groups, based on whether the children were recommended new glasses (prescription group, *n* = 61 (40%)) or not (control group, *n* = 91 (60%)). Out of the thirteen (9%) children who used glasses (for myopia), only one needed new glasses and was assigned to the prescription group. The other 60 children in the prescription group had not previously used glasses. The mean age ± SD was 15.2 ± 0.9 years (range 14–16 years, 41% boys) in the prescription group, and 15.1 ± 0.6 years (range 14–16 years, 40% boys) in the control group. There were no significant differences between the groups regarding age and sex.

All children had normal (control group) or correctable to normal (prescription group) vision and good ocular health, except two children who were referred to an ophthalmologist (peripheral retinal thinning, possible posterior staphyloma, blepharitis). In the study sample there were only small refractive errors (low myopia, low hyperopia), except for one participant with high myopia (SER − 5.00 D). Average cycloplegic refractive error in the prescription group was − 0.53 SER for the right eye, and the prescription group was significantly more myopic than the control group (SER, RE; t(150) = 5.750, *p* < 0.001) (Table 1). The prescription group also had significantly reduced habitual visual acuity compared with the control group (RE; t(58.146) = -15.093, *p* < 0.001). After correcting the refractive error there was no difference in visual acuity between the two groups (best corrected visual acuity). For binocular measurements, the binocular amplitude of accommodation was significantly lower in the prescription group (t(98.990) = 2.382, *p* = 0.019). Continuous reading/screen viewing ability was also significantly lower in the prescription group compared to the control group (χ^2^(1, *n* = 152) = 4.09, *p* = 0.043) (Table 2). Frequencies of visual function and symptom results are shown in Table 2. In the prescription group, 90% had uncorrected low myopia, and 10% had uncorrected low hyperopia with associated symptoms. In contrast, in the control group, 81% had emmetropia, 12% had fully corrected myopia, and 7% low hyperopia without associated symptoms. In the total sample, there were no significant differences between girls and boys regarding refractive error (myopia/hyperopia).

**Table 1:** Eye examination – average visual acuity, refractive error, and binocular status

		Prescription group (*n* = 61) Mean ± SD	Control group (*n* = 91) Mean ± SD
Habitual LogMAR VA (6 m)	RE	0.42 ± 0.21**	0.00 ± 0.02
	LE	0.42 ± 0.21**	0.01 ± 0.04
Best corrected logMAR VA (6 m)	RE	0.00 ± 0.03	0.00 ± 0.02
	LE	0.00 ± 0.02	-0.01 ± 0.04
Cycloplegic retinoscopy (SER) (6 m)	RE	-0.53 ± 0.52**	0.04 ± 0.65
	LE	-0.52 ± 0.53**	0.03 ± 0.62
Binocular vision measurements	Heterophoria 6 m	-0.13 ± 0.72	0.00 ± 0.30
	Heterophoria 40 cm	-3.41 ± 2.23	-3.35 ± 2.02
	NPC	7.80 ± 1.20	7.80 ± 0.80
	AA BIN	10.25 ± 1.79*	10.89 ± 1.26

VA: visual acuity, SER: spherical equivalent error in diopters (D), RE: right eye, LE: left eye, BIN: binocular (both eyes), NPC: near point of convergence (cm), AA: amplitude of accommodation (D), Heterophoria: negative values denote prism diopter exophoria, positive values prism diopter esophoria. Habitual VA data were missing for three children in the prescription group and 13 children in the control group. *Statistically significant difference between groups (*p* < 0.05). **Statistically significant difference between groups with Bonferroni adjusted alpha levels

**Table 2:** Sample frequencies of reported symptoms, vision results, and management

		Prescription group (n = 61)	Control group (n = 91)
		*n* (%)	*n* (%)
Symptoms; eye examination^a^	Blurred vision at distance	54 (89)**	7 (8)
	Headache	22 (36)	21 (23)
	No vision related symptoms	0 (0)**	66 (73)
Symptoms in general; questionnaireb	Eye pain	44 (72)	48 (53)
		2.6 ± 2.1	1.7 ± 1.9
	Headache	53 (87)	75 (82)
		3.8 ± 2.4	2.9 ± 2.1
	Neck pain	37 (61)	44 (48)
		1.9 ± 2.0	1.4 ± 1.8
	Shoulder pain	40 (66)**	39 (43)
		2.1 ± 2.0**	1.1 ± 1.5
	Lower back pain	34 (56)	39 (43)
		1.5 ± 1.8	1.4 ± 2.0
Symptoms during screen use; questionnaireb	Eye pain	47 (77)	63 (69)
		2.8 ± 2.1	2.3 ± 2.0
	Headache	51 (84)	74 (81)
		3.2 ± 2.3	3.1 ± 2.3
	Neck pain	43 (71)	49 (54)
		2.0 ± 1.6	1.5 ± 1.8
	Shoulder pain	40 (66)**	36 (40)
		1.9 ± 1.7**	1.0 ± 1.6
	Lower back pain	35 (57)	39 (43)
		1.6 ± 1.8	1.3 ± 1.8
Continuous reading/screen viewing ability		26 (43)*	54 (59)
Screen time (hours/day, mean ± SD)	Weekday (Sunday – Friday)	2.2 ± 1.4	2.0 ± 1.2
	Weekend (Saturday)	3.8 ± 2.2	4.1 ± 2.1
Ametropia (Cycloplegic SER, D)	Emmetropia (> -0.50 D, < +0.50 D)	0 (0)	74 (81)
	Low myopia (> -3.00 D, ≤ -0.50 D)	55 (90)	10 (11)
	High myopia (≤ -5.00 D)	0 (0)	1 (1)
	Low hyperopia ( ≥ + 0.50 D, ≤ +2.00 D)	6 (10)	6 (7)
	Anisometropia (≥ 1.00 D)	0 (0)	0 (0)
Hab VA RE (6 m)	LogMAR (≤ 0.0) *[decimal ≥ 1.0]*	6 (10)**	73 (80)
	LogMAR (≤ 0.1, > 0.0) *[decimal < 1.0, ≥ 0.8]*	1 (2)	5 (6)
	LogMAR (≤ 0.3, > 0.1) *[decimal < 0.8, ≥ 0.5]*	11 (18)**	0 (0)
	LogMAR (> 0.3) *[decimal < 0.5]*	40 (66)**	0 (0)
Best corrected VA RE (6 m)	LogMAR (≤ 0.0) *[decimal ≥ 1.0]*	51 (84)	85 (93)
	LogMAR (≤ 0.1, > 0.0) *[decimal < 1.0, ≥ 0.8]*	10 (16)	6 (7)
	LogMAR (≤ 0.3, > 0.1) *[decimal < 0.8, ≥ 0.5]*	0 (0)	0 (0)
	LogMAR (> 0.3) *[decimal < 0.5]*	0 (0)	0 (0)
Heterophoria (6 m)	Orthophoria	59 (97)	90 (99)
	Esophoria > 1 ΔBO	1 (2)	1 (1)
	Exophoria > 2 ΔBI	1 (2)	0 (0)
Heterophoria (40 cm)	Orthophoria	57 (93)	87 (96)
	Esophoria > 0 ΔBO	1 (2)	2 (2)
	Exophoria > 6 ΔBI	3 (5)	2 (2)
NPC (cm)	≤ 10	58 (95)	91 (100)
	> 10 < 25	3 (5)	0 (0)
AA BIN (D)	> 8	53 (87)**	90 (99)
	≤ 8	8 (13)**	1 (1)
Management - prescription of new glasses	Low myopia and blurred vision at distance	54 (89)	
	Low hyperopia and headache	6 (10)	
	Change of ≥ 0.50 best corrected SER (myopia)	1 (2)	

SER: spherical equivalent error in diopter (D), RE: right eye, VA: visual acuity, Hab: habitual, NPC: near point of convergence (cm), Heterophoria: prism diopter base in (ΔBI) or base out (ΔBO), AA: amplitude of accommodation (D), BIN: binocular (both eyes). ^a^None of the participants reported double vision, eye pain, or blurred vision at near. ^b^Symptoms in general and during screen use are reported as frequencies and average pain scores (mean ± SD) (Wong-Baker FACES Pain Rating Scale). Habitual VA data were missing for three children in the prescription group and 13 children in the control group. *Statistically significant difference between groups (*p* < 0.05). **Statistically significant difference between groups with Bonferroni adjusted alpha levels

Children in the prescription group reported significantly increased shoulder pain both in general and during screen use, compared to the control group (t(150) = -3.223, *p* = 0.002, t(150) = -3.303, *p* = 0.001) (Table 2). Children with shoulder pain had significantly reduced habitual visual acuity and lower continuous reading/screen viewing ability: Children experiencing shoulder pain in general (*n* = 79) (RE; 0.22 ± 0.27 vs. 0.13 ± 0.22 (mean ± SD), t(133.872) = -2.082, *p* = 0.039) and during screen use (*n* = 76) (RE; 0.25 ± 0.27 vs. 0.11 ± 0.21 (mean ± SD), t(129.038) = -3.219, *p* = 0.002) had reduced habitual visual acuity, and lower continuous reading/screen viewing ability (33/76 (43%) vs. 47/76 (62%), χ^2^(1, *n* = 152) = 5.17, *p* = 0.034) compared with the rest of the sample. More boys than girls reported using a computer (χ^2^(1, *n* = 152) = 6.14, *p* = 0.017), and boys in the control group had higher screen time during the weekend (Saturday) compared with girls (4.7 ± 2.0 vs. 3.7 ± 2.1 h/day (mean ± SD), t(89) = 2.299, *p* = 0.024). There were no significant sex differences regarding the use of smartphones and tablets. There were no significant correlations between shoulder pain, the use of different digital devices, and screen time. Eye pain, headache, and musculoskeletal pain were also analyzed by sex, and there were no overall significant differences between boys and girls. However, only boys in the prescription group had increased shoulder pain in general (t [58] = -3.901, *p* < 0.001) and during screen use (t(39.955) = -3.968, *p* < 0.001), compared with the control group.

**Fig. 2:**
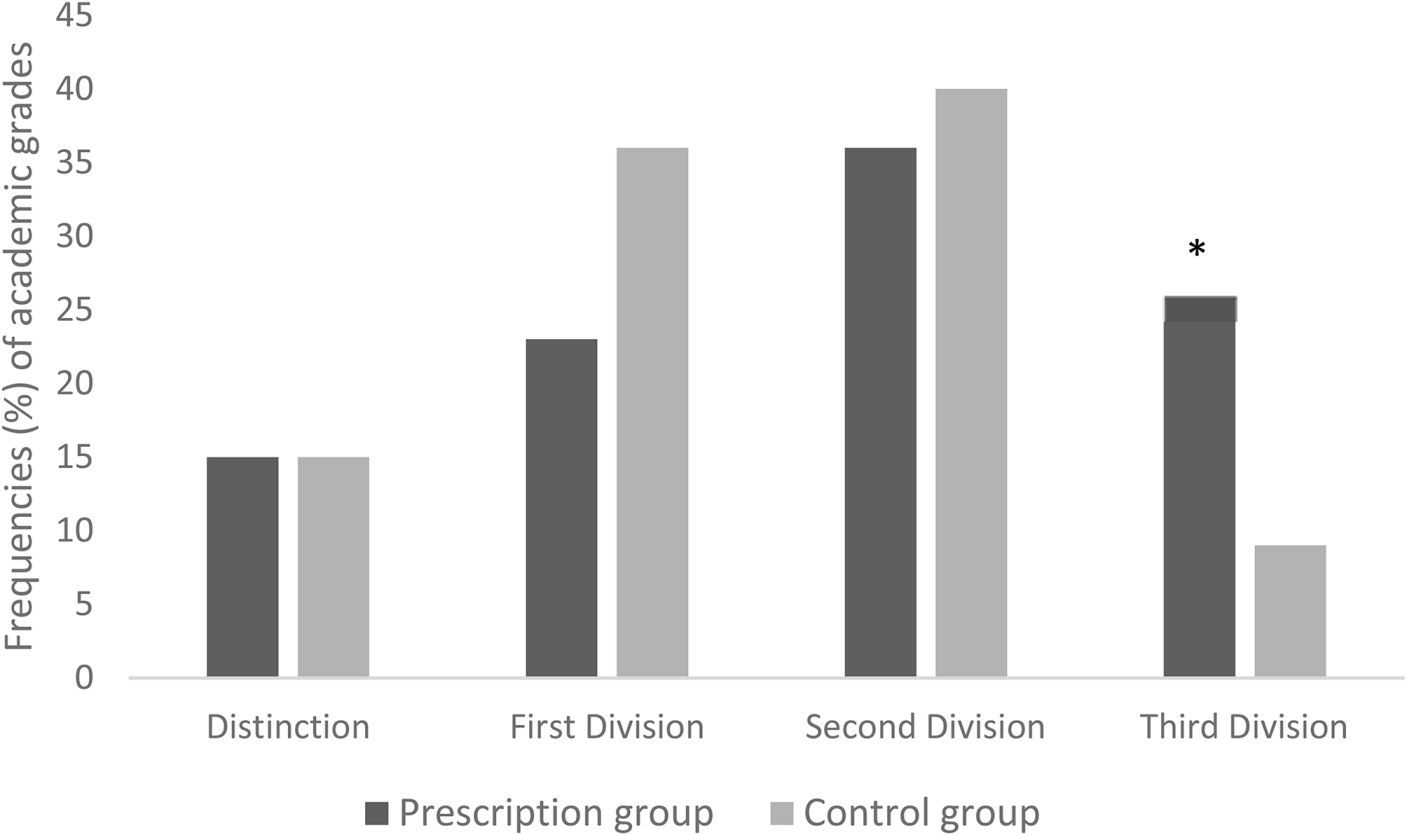
Sample frequencies (%) of academic grades in the prescription group (*n* = 61) and control group (*n* = 91). *Statistically significant difference between the groups (Bonferroni adjusted alpha level of 0.0125 (0.05/4))

There was a significant difference in academic scores between the prescription and control group (t(150) = -2.095, *p* = 0.038). Figure 2 shows sample frequencies of academic grades. In the prescription group, significantly more children had third division grades (26%) than in the control group (9%) (χ^2^(1, *n* = 152) = 8.35, *p* = 0.004). For the other grades, there were no significant differences between the groups.

Children with third division grades (*n* = 24) had reduced habitual visual acuity (RE; 0.38 ± 0.29 vs. 0.14 ± 0.23 (mean ± SD), t(134) = -4.193, *p* < 0.001), reduced binocular amplitude of accommodation (9.63 ± 1.41 vs. 10.82 ± 1.47 (mean ± SD), t(150) = 3.673, *p* < 0.001), and lower continuous reading/screen viewing ability (5/24 (21%) vs. 75/128 (59%), χ^2^(1, *n* = 152) = 11.56, *p* < 0.001) compared with the rest of the sample. There were no significant correlations between academic scores and screen time (all p’s > 0.05). Academic performance was also analyzed by sex, and only girls in the prescription group had a higher frequency of third division grades (χ^2^(1, *n* = 91) = 9.35, *p* = 0.006), compared with the control group. Overall, girls had near-significantly higher academic grades compared with boys (χ^2^(1, *n* = 152) = 7.49, *p* = 0.058).

## Discussion

In this study, reduced academic performance and increased musculoskeletal pain were significantly associated with uncorrected vision problems and reduced visual acuity. Sex and grade level significantly influenced the results. Adolescents with mainly uncorrected low myopia had lower overall academic grades at the nationwide final grade examination, and reduced habitual visual acuity was significantly associated with the reduced academic performance. One explanation is that the ability to see and follow what is presented at the blackboard/smartboard is important for the child’s learning. This is in line with a recent Chinese study with grade 7–9 students also reporting correlations between reduced visual acuity due to myopia and reduced overall academic performance [17]. Furthermore, in the present study, children with uncorrected vision had more third-division grades than children with normal vision/adequate prescribed eye wear, and this difference was only significant for girls. These results are in accordance with a recent cluster randomized clinical trial in the USA, where a total of 2304 grade 3–7 students received free eye examination and eyeglasses during one of three school years. The results showed a significant 1-year positive effect on reading scores in girls, not boys, and for students in special education and performing in the lowest quartile at baseline, before start of intervention. However, the impact was not sustained at a 2-year follow-up [24]. In another US study, educational outcomes after receiving eyeglasses in a free school-based vision program, were investigated in 406 low-income minority 1–5 grade students, compared to 23,393 school/grade peers. The results showed a 2-year positive impact on English language arts. In addition, students with baseline performance in the bottom tercile, had an immediate and sustained improvement in mathematics achievement [21]. These two studies and the present study point towards the notion that girls and children facing barriers to school performance, such as socioeconomic factors, will benefit the most from good vision care in schools [17, 18, 21, 24, 52]. In the present study, girls had a near-significant (*p* = 0.058) overall higher educational outcome than boys. Girls have been shown to have higher academic ambitions, and use more time on schoolwork and reading, compared with boys, and girls without optimal vision and lack of eyeglasses then have a disadvantage [11, 24, 53, 54].

Our results showed that the adolescents with uncorrected vision experienced more shoulder pain in general and during screen use compared with children with normal vision/adequate prescribed eye wear. When analysed by sex, this difference was only significant for boys. Shoulder pain was significantly associated with reduced visual acuity and lower continuous reading/screen viewing ability. These results are in line with two North European studies showing increased musculoskeletal pain and discomfort in children and adolescents with uncorrected vision problems and lack of proper eye wear [11, 28]. In one of these studies, children presenting with headache and upper body musculoskeletal pain, had more vision problems and lacked necessary corrective eye wear for near than a control group. Furthermore, musculoskeletal pain was significantly associated with reduced habitual VA [28]. In another North European study, children with the best visual acuity had less musculoskeletal pain during screen use than those with poorer visual acuity [35]. These associations may be explained by increased demand on the head-stabilizing musculature in the neck, shoulders, and back, thereby increasing the risk for pain symptoms [28, 29, 32, 33]. This indicates that correcting refractive errors may prevent and reduce musculoskeletal pain, an easy applicable and low-cost intervention.

In the present study, there were no significant correlations between musculoskeletal pain and screen use/time, except for the lower continuous reading/screen viewing ability in the children with shoulder pain during screen use. Interestingly, shoulder pain was increased only for boys in the prescription group, compared with the control group. This may be explained by sex differences in time used on different types of screens; however, the questionnaire lacked questions regarding time used on different digital devices. In the present study, more boys than girls reported using a computer, indicating higher computer use in boys. Previous studies have shown associations between upper body musculoskeletal pain symptoms, total screen time, time used on different types of digital screens (tablet, mobile), and screen viewing distance [11, 35, 55–59].

### Strengths and limitations

A strength of this study is that it provides new findings regarding the correlations between uncorrected vision problems, educational performance, and musculoskeletal pain symptoms, in adolescents in Asia. The findings agree with results from school children in the USA and Europe [11, 16, 17, 21, 24, 28, 35], elucidating the effects of uncorrected vision independent of factors such as genetics, culture, and socioeconomics. Another major strength is the use of cycloplegic refraction for evaluating refractive errors [60]. One limitation was that many of the children who participated in the school vision testing program were not participating in the study. The main reason was that participation required written consent from both parents, which was difficult to obtain, as many children lived far from home during school terms (e.g., boarding school) or long-term commuting parents. Several efforts were made to obtain both parents’ consent. This impacted the power in the subgroup analysis, however, the results were in line with larger studies [16, 17, 21, 24, 34]. The background information of the participating children is representative of the school population; however, one should be careful to generalize to prevalences. Symptoms and screen use were self-reported by the children, which may have biased the results. Time of use by different digital devices was not stratified, making it impossible to investigate symptoms related to the different screen types. It was planned to collect grades separately for different subjects. Unfortunately, we only got access to the final academic performance, averaged across subjects. This may represent a limitation of the study as different subjects have different visual requirements. Due to the nature of large school vision testing programs, involving several locations and personnel, there is a risk of missing data, compared to an assessment in an eye clinic. Another limitation of this study was the cross-sectional design, which makes it impossible to investigate causality between variables.

## Conclusions

This study showed significant associations between uncorrected vision problems and reduced academic performance and increased shoulder pain in school children. The children needing glasses had mainly low myopia, and reduced habitual visual acuity was significantly correlated with lower overall academic performance and more shoulder pain. Subanalyses showed that girls with uncorrected vision had lower academic performance, while boys with uncorrected vision had more musculoskeletal pain, compared with children with normal vision. This suggests that correcting even small refractive errors is important to optimize educational outcomes and prevent pain, and thereby increasing quality of life in adolescents.

## Data Availability

The dataset analysed during the current study is available from the corresponding author on reasonable request.

## References

[1] Marques AP, Ramke J, Cairns J, Butt T, Zhang JH, Jones I (2022). The economics of vision impairment and its leading causes: a systematic review. EClinicalMedicine.

[2] Purola P, Koskinen S, Uusitalo H (2023). Impact of vision on generic health-related quality of life - A systematic review. Acta Ophthalmol.

[3] Falkenberg HK, Langaas T, Svarverud E (2019). Vision status of children aged 7–15 years referred from school vision screening in Norway during 2003–2013: a retrospective study. BMC Ophthalmol.

[4] Hagen LA, Gilson SJ, Baraas RC (2020). Vision status and reading test results in adolescents in Norway. Scandinavian J Optometry Visual Sci.

[5] Junghans B, Kiely PM, Crewther DP, Crewther SG (2002). Referral rates for a functional vision screening among a large cosmopolitan sample of Australian children. Ophthalmic Physiol Opt.

[6] Bodack MI, Chung I, Krumholtz I (2010). An analysis of vision screening data from New York City public schools. Optometry.

[7] White SLJ, Wood JM, Black AA, Hopkins S (2017). Vision screening outcomes of Grade 3 children in Australia: differences in academic achievement. Int J Educ Res.

[8] Mahayana IT, Indrawati SG, Pawiroranu S (2017). The prevalence of uncorrected refractive error in urban, suburban, exurban and rural primary school children in Indonesian population. Int J Ophthalmol.

[9] Sanchez-Gonzalez MC, Palomo-Carrion R, De-Hita-Cantalejo C, Romero-Galisteo RP, Gutierrez-Sanchez E, Pinero-Pinto E (2022). Visual system and motor development in children: a systematic review. Acta Ophthalmol.

[10] Hopkins S, Black AA, White SLJ, Wood JM (2019). Visual information processing skills are associated with academic performance in Grade 2 school children. Acta Ophthalmol.

[11] Thorud HS, Mork R, Bjorset CO, Gilson SJ, Hagen LA, Langaas T (2022). Laboured reading and musculoskeletal pain in school children - the role of lifestyle behaviour and eye wear: a cross-sectional study. BMC Pediatr.

[12] Hopkins S, Narayanasamy S, Vincent SJ, Sampson GP, Wood JM (2020). Do reduced visual acuity and refractive error affect classroom performance?. Clin Exp Optom.

[13] Mavi S, Chan VF, Virgili G, Biagini I, Congdon N, Piyasena P (2022). The impact of Hyperopia on Academic Performance among children: a systematic review. Asia Pac J Ophthalmol (Phila).

[14] UptoDate. Refractive errors in children [updated June 1. 2023. Available from: https://www.uptodate.com/contents/refractive-errors-in-children?search=refractive%20error&source=search_result&selectedTitle=1~58.

[15] Ntodie M, Saunders K, Little JA (2023). Accuracy and stability of accommodation and vergence responses during sustained near tasks in uncorrected hyperopes. Sci Rep.

[16] Bruce A, Kelly B, Chambers B, Barrett BT, Bloj M, Bradbury J (2018). The effect of adherence to spectacle wear on early developing literacy: a longitudinal study based in a large multiethnic city, Bradford, UK. BMJ Open.

[17] Jan C, Li SM, Kang MT, Liu L, Li H, Jin L (2019). Association of visual acuity with educational outcomes: a prospective cohort study. Br J Ophthalmol.

[18] Ma X, Zhou Z, Yi H, Pang X, Shi Y, Chen Q (2014). Effect of providing free glasses on children’s educational outcomes in China: cluster randomized controlled trial. BMJ.

[19] Harvey EM, Miller JM, Twelker JD, Davis AL (2016). Reading fluency in school-aged children with bilateral astigmatism. Optom Vis Sci.

[20] Narayanasamy S, Vincent SJ, Sampson GP, Wood JM (2015). Simulated astigmatism impairs academic-related performance in children. Ophthalmic Physiol Opt.

[21] Dudovitz RN, Sim MS, Elashoff D, Klarin J, Slusser W, Chung PJ (2020). Receipt of corrective lenses and academic performance of low-income students. Acad Pediatr.

[22] Glewwe P, West KL, Lee J (2018). The impact of providing Vision Screening and Free eyeglasses on Academic outcomes: evidence from a Randomized Trial in Title I Elementary schools in Florida. J Policy Anal Manage.

[23] Ma Y, Congdon N, Shi Y, Hogg R, Medina A, Boswell M (2018). Effect of a Local Vision Care Center on eyeglasses Use and School Performance in Rural China: a Cluster Randomized Clinical Trial. JAMA Ophthalmol.

[24] Neitzel AJ, Wolf B, Guo X, Shakarchi AF, Madden NA, Repka MX (2021). Effect of a Randomized Interventional School-based Vision Program on Academic performance of students in grades 3 to 7: a Cluster Randomized Clinical Trial. JAMA Ophthalmol.

[25] Akinci A, Guven A, Degerliyurt A, Kibar E, Mutlu M, Citirik M (2008). The correlation between headache and refractive errors. J AAPOS.

[26] Dotan G, Stolovitch C, Moisseiev E, Cohen S, Kesler A (2014). Uncorrected amteropia among children hospitalized for headache evaluation: a clinical descriptive study. BMC Pediatr.

[27] Hendricks TJ, van Der Horst JDEB, Hendrikse FG, Knottnerus F (2007). Relationship between habitual refractive errors and headache complaints in schoolchildren. Optom Vis Sci.

[28] Thorud HS, Aurjord R, Falkenberg HK (2021). Headache and musculoskeletal pain in school children are associated with uncorrected vision problems and need for glasses: a case-control study. Sci Rep.

[29] Sanchez-Gonzalez MC, Gutierrez-Sanchez E, Sanchez-Gonzalez JM, Rebollo-Salas M, Ruiz-Molinero C, Jimenez-Rejano JJ (2019). Visual system disorders and musculoskeletal neck complaints: a systematic review and meta-analysis. Ann N Y Acad Sci.

[30] Blehm C, Vishnu S, Khattak A, Mitra S, Yee RW (2005). Computer vision syndrome: a review. Surv Ophthalmol.

[31] Rosenfield M (2011). Computer vision syndrome: a review of ocular causes and potential treatments. Ophthalmic Physiological Optics: J Br Coll Ophthalmic Opticians.

[32] de Vries J, Ischebeck BK, Voogt LP, Janssen M, Frens MA, Kleinrensink GJ (2016). Cervico-ocular Reflex is increased in people with nonspecific Neck Pain. Phys Ther.

[33] Johnston JL, Daye PM, Thomson GT (2017). Inaccurate saccades and enhanced Vestibulo-Ocular Reflex suppression during Combined Eye-Head movements in patients with chronic Neck Pain: possible implications for cervical Vertigo. Front Neurol.

[34] Lajmi H, Choura R, Ben Achour B, Doukh M, Amin Z, Hmaied W (2021). Headache associated with refractive errors: characteristics and risk factors. Rev Neurol (Paris).

[35] Falkenberg HK, Johansen TR, Schiøtz Thorud HM. Headache, eyestrain, and musculoskeletal symptoms in relation to smartphone and tablet use in healthy adolescents. Scandinavian J Optometry Visual Sci. 2020;13(2).

[36] Mahmoud NF, Hassan KA, Abdelmajeed SF, Moustafa IM, Silva AG (2019). The relationship between Forward Head posture and Neck Pain: a systematic review and Meta-analysis. Curr Rev Musculoskelet Med.

[37] Global GBD (2018). Global, regional, and national incidence, prevalence, and years lived with disability for 354 diseases and injuries for 195 countries and territories, 1990–2017: a systematic analysis for the global burden of Disease Study 2017. Lancet.

[38] Gustafsson ML, Laaksonen C, Aromaa M, Loyttyniemi E, Salantera S (2018). The prevalence of neck-shoulder pain, back pain and psychological symptoms in association with daytime sleepiness - a prospective follow-up study of school children aged 10 to 15. Scand J Pain.

[39] Joergensen AC, Hestbaek L, Andersen PK, Nybo Andersen AM (2019). Epidemiology of spinal pain in children: a study within the Danish National Birth Cohort. Eur J Pediatr.

[40] GBD 2019. Global burden of 369 diseases and injuries in 204 countries and territories, 1990–2019: a systematic analysis for the global burden of Disease Study 2019. Lancet. 2020;396(10258):1204–22. 10.1016/S0140-6736(20)30925-9 PMC7567026 33069326

[41] American Optometric Association. Comprehensive Pediatric Eye and Vision Examination 2017 [Available from: https://www.aoa.org/optometrists/tools-and-resources/clinical-care-publications/clinical-practice-guidelines?sso=y.

[42] College of Optometrists. The routine eye examination 2022 [Available from: https://guidance.college-optometrists.org/guidance-contents/knowledge-skills-and-performance-domain/the-routine-eye-examination/.

[43] Nepal EH. Nepalese Association Of Optometrist (NAO) 2021 [Available from: https://www.eyehealthnepal.com/nepalese-association-of-optometrist-nao/.

[44] Neely JC (1956). The R.A.F. near-point rule. Br J Ophthalmol.

[45] Holden BA, Fricke TR, Wilson DA, Jong M, Naidoo KS, Sankaridurg P (2016). Global prevalence of myopia and high myopia and temporal trends from 2000 through 2050. Ophthalmology.

[46] American Academy of Ophthalmology. Hyperopia 2023 [Available from: https://eyewiki.org/Hyperopia.

[47] O’Donoghue L, Saunders KJ, McClelland JF, Logan NS, Rudnicka AR, Gilmartin B (2010). Sampling and measurement methods for a study of childhood refractive error in a UK population. Br J Ophthalmol.

[48] Hagen LA, Gjelle JVB, Arnegard S, Pedersen HR, Gilson SJ, Baraas RC (2018). Prevalence and possible factors of myopia in Norwegian adolescents. Sci Rep.

[49] Scheiman M, Wick B. Clinical management of Binocular Vision: Heterophoric, Accommodative, and Eye Movement disorders. 4th ed. Lippincott Williams & Wilkins; 2013.

[50] Wong-Baker FACES, Foundation. Wong-Baker FACES® Pain Rating Scale 2016 [Available from: https://wongbakerfaces.org/.

[51] Altman DG. Practical statistics for medical research. Chapman & Hall; 1991.

[52] Slavin RE, Collins ME, Repka MX, Friedman DS, Mudie LI, Owoeye JO (2018). In Plain Sight: reading outcomes of providing eyeglasses to Disadvantaged Children. J Educ Students Plac.

[53] Sikora J, Saha LJ (2009). Gender and professional career plans of high school students in comparative perspective. Educational Res Evaluation.

[54] Wicht A, Miyamoto A, Lechner CM (2021). Are girls more ambitious than boys? Vocational interests partly explain gender differences in occupational aspirations. J Career Dev.

[55] Costigan SA, Barnett L, Plotnikoff RC, Lubans DR (2013). The health indicators associated with screen-based sedentary behavior among adolescent girls: a systematic review. J Adolesc Health.

[56] Joergensen AC, Strandberg-Larsen K, Andersen PK, Hestbaek L, Andersen AN (2021). Spinal pain in pre-adolescence and the relation with screen time and physical activity behavior. BMC Musculoskelet Disord.

[57] Myrtveit SM, Sivertsen B, Skogen JC, Frostholm L, Stormark KM, Hysing M (2014). Adolescent neck and shoulder pain–the association with depression, physical activity, screen-based activities, and use of health care services. J Adolesc Health.

[58] Szita J, Boja S, Szilagyi A, Somhegyi A, Varga PP, Lazary A (2018). Risk factors of non-specific spinal pain in childhood. Eur Spine J.

[59] Torsheim T, Eriksson L, Schnohr CW, Hansen F, Bjarnason T, Valimaa R (2010). Screen-based activities and physical complaints among adolescents from the nordic countries. BMC Public Health.

[60] Bjørset CO, Pedersen HR, Synstelien GO, Gilson SJ, Hagen LA, Langaas T (2022). Non-cycloplegic refraction cannot replace cycloplegic refraction when screening for refractive errors in children. Scandinavian J Optometry Visual Sci.

